# Nursing Students’ Knowledge and Attitudes toward Danger Signs in Neonatal Illnesses

**DOI:** 10.3390/medicina59111939

**Published:** 2023-11-01

**Authors:** Ebtihal Eltyeb, Randa Basheer, Amel Ahmed, Ali Al-Makramani, Mohamed Salih Mahfouz, Amna Mohamedali, Anwar Balla, Halima Algadi, Gassem Gohal

**Affiliations:** 1 Faculty of Medicine, Jazan University, Jazan P.O. Box 114, Saudi Arabia; makra3@yahoo.com (A.A.-M.); mm.mahfouz@gmail.com (M.S.M.); dr.gassem@gmail.com (G.G.); 2 Faculty of Nursing, Jazan University, Jazan P.O. Box 114, Saudi Arabia; randaaby7@gmail.com (R.B.); amel09180@gmail.com (A.A.); amna.ahmed45@yahoo.com (A.M.); nonabala366@gmail.com (A.B.); halimaalgadi1900@gmail.com (H.A.)

**Keywords:** attitude, knowledge, danger signs, neonatal illness, nursing students

## Abstract

*Background and Objectives*: Neonates can show subtle signs of illness that could be overlooked by their mothers and caregivers. Therefore, basic knowledge regarding neonatal health and early detection of neonatal diseases can help improve survival. We assessed nursing students’ knowledge and attitudes toward the danger signs of neonatal illnesses. *Materials and Methods*: We performed a descriptive cross-sectional study using a structured online questionnaire based on danger signs categorized by the World Health Organization to assess knowledge of neonatal illness danger signs among nursing students. *Results*: We assessed 342 students, of which 67.0% (95% CI: 61.8–71.7) had good knowledge regarding neonatal illness danger signs, and 71.6% received information about neonatal care. About 33% of the participants had a basic knowledge of neonatal care, such as breastfeeding, immunization, routine postnatal care, and eye care. Students who received information on neonatal care were three times (AOR = 2.95, 95% CI: 11.59–5.47, *p* = 0.001) more likely to have good knowledge than those who did not. The students also showed a positive attitude towards the importance of knowledge regarding neonatal illness danger signs, teaching this subject in their college, participating in studies involving the practice and knowledge of mothers regarding neonatal illness danger signs, and the importance of knowledge in reducing neonatal mortality in the region. *Conclusions*: More training programs on neonatal care should be implemented to enhance knowledge and raise nursing students’ awareness of the dangerous signs of neonatal illnesses.

## 1. Introduction

Despite increasing efforts to counteract neonatal morbidity and mortality, it remains a significant health concern for communities and governments. The World Health Organization (WHO) reported that 5.3 million children under five had died globally, and approximately 50% died within 28 days after birth [1]. The neonatal period is the first 28 days post-birth and is regarded as the most vulnerable period, often involving preterm delivery problems, intrapartum-related difficulties, and newborn sepsis, which are the leading causes of neonatal deaths worldwide [2].The WHO defines“neonatal death”as a death that occurs within the first 28 days post-birth [3]. There have been notable improvements in children and adolescent health anddecreasedchild mortality in Saudi Arabia; however, the neonatal mortality rate is a major concern for health authorities. According to the General Authority for Statistics reported in 2016, the Jazan region, the third largest region in Saudi Arabia, has a higher neonatal mortality rate than other regions [4].

The WHO categorizes danger signs in neonates as follows: no initiation of breastfeeding since birth or stopped feeding;seizures; fast breathing rate; shortness of breath (tachypnea); skin temperature greater than 37.5 °C (hyperthermia) or below 35.5 °C (hypothermia); weakness or lethargy; yellow soles (may indicate jaundice); signs of local infection (umbilicus pus draining or redness, skin boils, or eyes draining pus) [5]. Many neonatal fatalities can be attributed to delays in appropriate health management. Initial delays are caused by parents who cannot recognize fatal manifestations of neonatal illness, followed by poor access to proper health facilities, and finally by healthcare workers who delay diagnosis and appropriate treatment for neonatal illnesses [6,7,8]. Therefore, awareness of neonatal illness danger signs is essential for healthcare workers, and it can significantly decrease neonatal morbidity and mortality.

As future healthcare workers, nursing students play a critical role in educating parents and caregivers about neonatal illness danger signs before delivery and in providing optimal care for neonates post-delivery. Therefore, this study evaluated the knowledge and attitudes of nursing students regarding dangerous signs of neonatal illness in the Jazan region.

## 2. Materials and Methods

### 2.1. Study Design and Area

We conducted a descriptive cross-sectional study between September 2022 and November 2022 in Jazan, Saudi Arabia. The Jazan region is the smallest in the Kingdom and has the highest population density; it has a total land area of approximately 11,671 km^2^ and a population of approximately 1.8 million. The area is divided into three distinct zones: the coastal plain, the highland, and the fertile plains, with numerous villages (rural areas) and towns (urban areas) in these zones.

### 2.2. Participants

We collected data from nursing students in the College of Nursing at Jazan University. The nursing colleges are located in four towns in the Jazan region, with the main campus in Jazan City and the other three in Farasan, Deir, and Darb. The university has a four-year nursing program consisting of two semesters (levels) per year and one year of training (internship). The curriculum of the third year of the course includes subspecialty modules, such as pediatrics, surgery, and obstetrics. However, in their second year of college, students generally receive information regarding the care needed for patients in various age groups. Female students constituted the majority of the participants; male students studied only at the main campus in Jazan. The inclusion criteria were as follows: all nursing students (male and female) in their second, third, fourth, and internship years who agreed to participate and complete the form. The sample size of this study was estimated to be 385 using the Cochran formula: *n* = (z) 2 p (1 − p)/d2,wherepisthe prevalence of knowledge at 50%, Z is 95% confidence interval, and d is an error of not more than 4% [9].

### 2.3. Data Collection Tool

We prepared a study questionnaire based on the WHO neonatal illness danger signs criteria and a thorough literature review. Then, we conducted a pilot study with 20 students who answered our questionnaire to assess the comprehensiveness of each question, answer, non-response, and response time. The questionnaire included an informed written consent section and demographic data, including sex, residence, year of study, and marital status. Furthermore, there were general knowledge questions assessing the definition of the neonate, whether the student had received any information about neonatal danger signs before, the type of information in the neonatal care they knew, and whether they had participated in or practiced any neonatal care before. The second section explored the participant’s knowledge of neonatal illness danger signs based on the danger signs categorized by WHO as follows: no initiation of feeding since birth or eating disorders; convulsion; respiratory rate of 60 or more (tachypnea); severe chest in-drawing (dyspnea); body temperature ≥37.5 °C (hyperthermia) or ≤35.5 °C (hypothermia); weakness or lethargy; yellow soles (may indicate jaundice); and umbilical infection, skin boils, or eyes draining pus (signs of local infection) [5]. The WHO signs were distributed into four main domains (general, respiratory, digestive, and nervous system signs). Each domain contained WHO sign options in addition to other non-related options. The last part of the questionnaire used a five-point Likert scale, exploring the student’s perspective towards the importance of awareness of neonatal illness danger signs for nursing students, their opinions regarding teaching this topic in the nursing college, their views regarding researching neonatal illness danger signs involving mother and caregiver practice and knowledge, and their thoughts on the role of knowledge in lowering neonatal mortality in the Jazan region. We used a non-probability convenience sampling method to recruit the participants. We collected data from participants who were accessible with no further requirements except for the availability and interest of the respondents to participate.

### 2.4. Data Presentation and Statistical Analysis

All data were analyzed using SPSS version 23.0 (IBM Corp, Armonk, NY, USA). All the information was initially gathered through a questionnaire and coded as variables. For the reliability analysis of the questionnaire, Cronbach’s Alpha test was used (α = 0.8769). The Kaiser–Meyer–Olkin Measure was used to determine sample adequacy (KMO = 0.827), which demonstrated a correlation between items of high magnitude. Bartlett’s Test of Sphericity was statistically significant at *p* = 0.000, allowing us to assume sphericity. Varimax rotation was used to ensure internal consistency. Descriptive and inferential statistics involving the Pearson chi-square test assessed the association between the knowledge score and different variables.A one-way analysis of variance (ANOVA) determined the differences between the means of two or more independent groups and knowledge scores. Binary logistic regression was used, with the dependent variable having only two categories, to predict whether these variables influenced the knowledge score. ANOVA effect sizes were calculated using eta-squared (η^2^), while Cohen’s d effect size was used for independent-sample *t*-tests. Effect sizes for Cohen’s d were classified as no effect (0–0.19), small (0.20–0.49), moderate (0.50–0.79), and large (≥0.8) to determine the magnitude of differences. The eta-squared (η^2^) effect size was considered small (0.01–0.06), medium (0.06–0.14), and large (>0.14). A *p*-value less than 0.05 was considered statistically significant.

### 2.5. Score

The questionnaire consisted of 21 questions that evaluated knowledge of neonatal illness danger signs. The first question was a multiple-choice question and asked about the definition of a neonate. The remaining questions were divided into four domains, each containing five yes or no questions related to signs of neonatal danger. For each domain, one point was given for each correct answer, resulting in a score range of 0–5, which was then converted into a percentage. The attitude was measured using a five-point Likert scale, with one indicating less importance and five indicating greater importance. The average score of the four attitude questions was converted into a percentage using the formula (Mean − 1) × 25. A student’s knowledge level was considered good if they scored above 50%, indicating that they had identified more than half of the neonatal danger signs mentioned.

## 3. Results

A total of 342 students were interviewed, with a response rate of 88.8% (342 out of 400). Table 1 describes the respondents’ socio-demographic characteristics. Most participants were female (83.0%), and most were unmarried/single (80.7%). About 57.3% of participants were in their fourth year of study, and 53.2% lived in Jazan town. We found that 57.9% of the participants learned about danger signs in neonatal illness, and 71.6% received information about neonatal care (Table 1).

**Figure 1:**
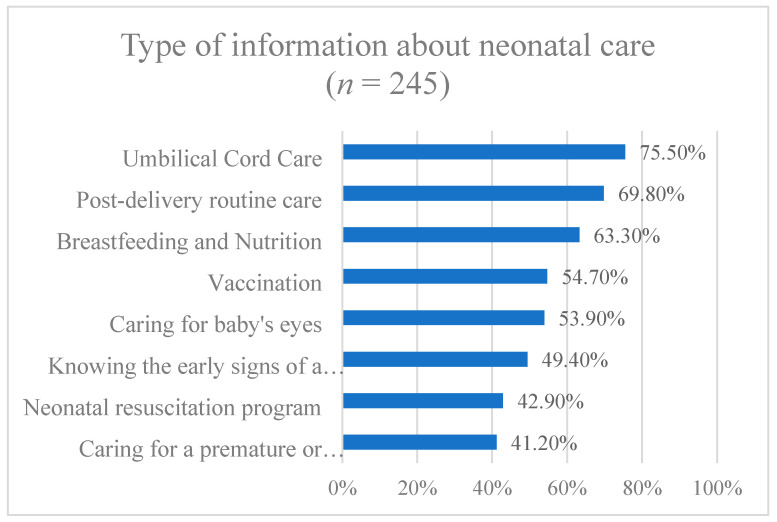
Students’ knowledge regarding types of information received about neonatal care.

As shown in Figure 1, over half of the participants had basic knowledge of neonatal care, such as umbilical cord care, breastfeeding and nutrition, vaccination, post-delivery routine care, and neonatal eye care.

Table 2 shows the magnitude of students’ knowledge regarding the definition of neonates and the signs of danger of neonatal illness. Results showed that 60% of the students knew the correct definition of a neonate, with widely different responses regarding the danger signs of neonatal illness. About 42.7% of students knew that hyperthermia was one of the main signs of neonatal danger, while more than 57.6% reported convulsions as one of the prominent signs of neonatal danger. However, the responses regarding neonatal danger signs related to the respiratory and digestive systems varied remarkably.

Table 3 shows the level of knowledge regarding neonatal illness danger signs among the study participants according to the selected characteristics. About 229 (67.0%; 95% CI: 61.8–71.7) students had good knowledge of neonatal danger signs. Male participants showed a higher level of knowledge (74.1%) than female participants, but the difference was not statistically significant (*p* = 0.202). Furthermore, those with previous knowledge about danger signs in neonatal illness (70.7%), those who practiced any neonatal care (67.7%), and those who received knowledge about the management of very ill neonates (69.1%) showed a higher level of knowledge compared with those who did not.However, the difference was not statistically significant (*p* = 0.38). Students’ knowledge was significantly affected by receiving information on neonatal care (*p* = 0.001), as shown in Table 3. The other variables in the table did not show any statistically significant associations with the level of knowledge (*p* > 0.05). 

Table 4 indicates that participants who received information on neonatal care had significantly higher scores for neonatal danger signs than those who did not, with a medium effect size (t = 17.20; *p* < 0.001, d = 0.50). The mean knowledge scores also differed significantly according to marital status, with a small effect size (F = 2.55; *p* = 0.039; η^2^ = 0.029).

Table 5 illustrates the factors associated with students’ knowledge of neonatal illness danger signs based on the logistic regression model.Receiving information on neonatal care was a factor significantly associated with students’ good knowledge of neonatal illness danger signs, as those who received the knowledge were three times (AOR = 2.95, 95% CI: 11.59–5.47, *p* = 0.001) more likely to be knowledgeable than those not receiving knowledge.

**Figure 2:**
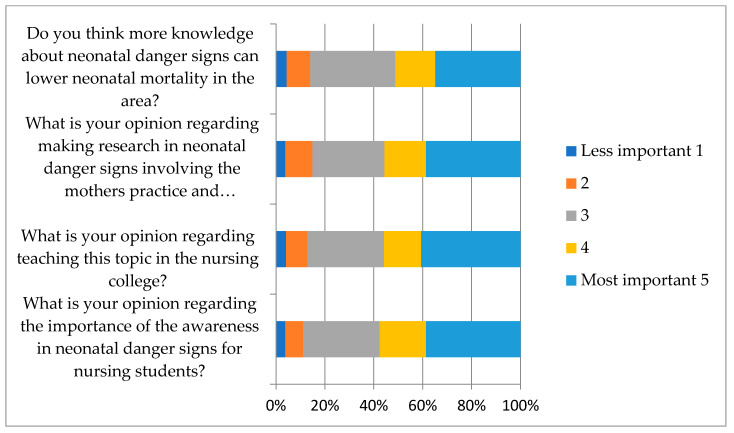
Students’ attitude regarding neonatal danger signs.

The responses related to the four questions regarding attitude showed that about half of the students had a good attitude towards the importance of the knowledge of neonatal danger signs for nursing students, their opinions regarding teaching this topic in the nursing college, their opinions regarding researching neonatal danger signs involving mothers’ and caretakers’ practice and knowledge in the Jazan region, and their thoughts on the role of knowledge in lowering neonatal mortality in the area. Figure 2 shows the distribution of students’ responses to the questions on attitude using the Likert scale.

## 4. Discussion

Three major delays contributing to neonatal death are related to the decisions to seek, care for, and receive proper care. The decision to seek care was the first of three delays that significantly influenced neonatal death, which was related to mothers’ and caregivers’ awareness, whereas receiving care is considered the third and is related to healthcare professionals’ knowledge of signs of neonatal danger [8]. Therefore, most studies have assessed mothers’ awareness regarding neonatal illness danger signs, significantly contributing to lower neonatal mortality and helping raise awareness among mothers and caretakers regarding seeking early medical care [10,11,12]. However, the third delay, receiving proper medical care, is vital in lowering neonatal mortality and is the least studied. Nursing students are essential healthcare professionals because they interact personally with patients, contribute to their education and counseling, and provide optimal care to neonates.

This study assessed the knowledge of nursing students at Jazan University in Southern Saudi Arabia regarding the dangerous signs of neonatal illness. Data analysis showed that more than half of the students adequately knew the neonatal danger signs. Furthermore, the most critical determinants of good knowledge were previously gained knowledge in neonatal care and marital status. Almost 70% of the students reported receiving education regarding neonatal care at their college, although only 60% knew the correct definition of the term “neonate”. Prior knowledge and receiving information about neonatal care strongly correlated with a greater understanding of neonatal danger signs; therefore, students who did not receive any information regarding neonatal care lacked knowledge and had a 2.7-fold greater likelihood of being unaware of neonatal danger signs than those who were taught.

Nonetheless, more than half of the students reported receiving information on umbilical cord clamping, post-delivery routine care, breastfeeding, and vaccination. Moreover, 49% and 43% of participants stated that they knew the early signs of neonatal illness and resuscitation, respectively, as shown in Figure 1, and 77% reported practicing neonatal care during their college studies. Our study revealed that marital status was significantly positively associated with students’ knowledge of neonatal illness danger signs, consistent with a study conducted among mothers in China [13]. This could be attributed to married people being more likely to be aware of the danger signs of neonatal illness through experience and practice.

As demonstrated in this study, the three most commonly known danger signs for students were jaundice (59.9%), convulsions (57.6%), and fever (55.3%). Because these symptoms are clear indicators, mothers or healthcare professionals do not commonly miss them. Numerous studies partly agree with this finding, indicating that fever was among the most frequently reported danger signs [14,15,16]. However, some danger signs in neonatal illness are easily missed because they may be subtle or unclear, such as some forms of neonatal convulsions, hypothermia, and signs of local infections, while some may be difficult to interpret, such asreluctance to feed and lethargy, which are attributed primarily to mothers’ normal neonatal behavior and are related to prolonged sleep [13,17,18]. In another study in Saudi Arabia that included 1428 female participants, only three or more danger signs were mentioned by 30% of the participants. Yellow soles, not feeding from birth or ceasing to feed, and local infection symptoms were the most frequently reported danger signs that participants were aware of; however, approximately two-thirds reported having encountered at least one of these warning indicators in their infants [10].

Many studies have adopted the WHO approach of diagnosing and managing severe diseases in infants under two months old in response to the need for improved illness detection in the postnatal period [19,20,21]. As a result, it is essential to assess the knowledge of neonatal illness danger signs among healthcare professionals who frequently interact with neonates and to adopt the WHO approach, which involves a significant proportion of healthcare workers at all career stages, including students and those in their early years of practice.

The general attitudes of the students regarding the perception of neonatal illness danger signs showed good scores, as most agreed with the importance of knowledge on this vital topic for nursing students, playing an essential role in lowering neonatal mortality. Moreover, many participants showed a positive attitude toward studies that can help improve mothers’ and caretakers’ knowledge and practice regarding neonatal illness danger signs in the Jazan region. We believe this study can help identify knowledge gaps and revise the university curriculum to better train nursing students who will be future healthcare workers. However, this study had some limitations. As the questionnaire was conducted online, there was a risk of desirability bias. Furthermore, we utilized a convenience sampling design, which may have limited reproducibility and generalizability.

## 5. Conclusions

This study determined the knowledge of neonatal illness danger signs among nursing students at Jazan University in Saudi Arabia. The study showed that nursing students had adequate knowledge regarding neonatal illness danger signs. However, one critical factor significantly influencing an adequate knowledge score is previous education and neonatal care training. Therefore, adequate training, supervision, and active community involvement in neonatal care should be implemented in the program curriculum to reinforce knowledge and raise awareness among students toward neonatal danger signs.

## Figures and Tables

**Table 1:** The characteristics of the participant students (*n* = 342).

Demographic Characteristics	Number	Percent (%)
Gender		
Male	58	17.0
Female	284	83.0
Year of study in the faculty of nursing		
2nd year	12	3.5
3rd year	104	30.4
4th year	196	57.3
Internship year	30	8.8
Marital status		
Divorced	6	1.8
Widow	15	4.4
Married without children	22	6.4
Married with children	23	6.7
Single	276	80.7
Area of residence		
Jazan(urban)	182	53.2
Jazan (rural)	103	30.1
Other towns out of the Jazan region	57	16.7
Previous knowledge about danger signs in neonatal illness		
Yes	198	57.9
No	144	42.1
Received any information about neonatal care		
Yes	245	71.6
No	97	28.4
Source of received information about neonatal care		
In the university	219	89.4
Outside the university	26	10.6
Type of information about neonatal care (*n* = 245)		
Caring for a premature or underweight baby	101	41.2
Neonatal resuscitation program	105	42.9
Knowing the early signs of a child’s illness	121	49.4
Caring for baby’s eyes	132	53.9
Vaccination	134	54.7
Breastfeeding and Nutrition	155	63.3
Post-delivery routine care	171	69.8
Umbilical Cord Care	185	75.5
Practice in any neonatal care during college training		
Yes	266	77.8
No	76	22.2
Recall an ill neonate personally with any of the neonatal danger signs during study or practice		
Yes	161	47.1
No	181	52.9
Recall the outcome (*n* = 161)		
Live with poor outcome	20	12.4
Do not know	34	21.1
Live with good outcome	40	24.8
Neonatal death	67	41.6
Previous knowledge about the management of the very ill neonate		
Yes	178	52.0
No	164	48.0

**Table 2:** Students’ knowledge regarding the definition of neonate and the danger signs in neonatal illness (*n* = 342).

Knowledge Variables	Number	Percent (%)
Definition of neonate		
Under 12 months of age	20	5.8
Do not know	36	10.5
Under three months of age	73	21.3
Under 28 days of age	213	62.3
Neonatal danger sign		
Urinate 12 h after birth	106	31.0
Not feeding since birth or stopped feeding	135	39.5
Hypothermia (temperature ≤ 35.5 °C)	146	42.7
Sign of local infection (umbilicus infection, skin boils, or eye infection)	168	49.1
Overweight baby	185	54.1
Fever (temperature of ≥ 37.5 °C)	189	55.3
Neonatal danger signs related to the respiratory system		
Blocked nose	122	35.7
Cough	123	36.0
Fast breathing (respiratory rate of 60 or more)	159	46.5
Severe chest in drawing (difficulty in breathing)	165	48.2
Slow breathing (respiratory rate less than 40)	230	67.3
Neonatal danger signs related to the digestive system		
Regurgitation after feeding	109	31.9
Two to three loose stools per day	117	34.2
Infantile colic	148	43.3
Constipation	171	50.0
Yellow soles (a sign of jaundice)	205	59.9
Neonatal danger signs related to the nervous system		
Unable to roll over	99	28.9
Crying	100	29.2
Closed hands	132	38.6
Weakness or lethargy	169	49.4
Convulsions	197	57.6

**Table 3:** Level of knowledge regarding neonatal danger signs among study participants according to some selected characteristics (*n* = 342).

Characteristic		Knowledge Level				*p* Value
		Poor		Good		
		N	%	N	%	
Gender	Male	15	(25.9)	43	(74.1)	0.202
	Female	98	(34.5)	186	(65.5)	
Year of study in the faculty of nursing	2nd year	2	(16.7)	10	(83.3)	0.648
	3rd year	35	(33.7)	69	(66.3)	
	4th year	65	(33.2)	131	(66.8)	
	Internship year	11	(36.7)	19	(63.3)	
Marital status	Non married	123	(44.6)	153	(55.4)	0.279 #
	Married with children	12	(52.2)	11	(47.8)	
	Married without children	11	(50.0)	11	(50.0)	
	Divorced	5	(83.3)	1	(16.7)	
	Widow	9	(60.0)	6	(40.0)	
Residence	Jazan region	90	(31.6)	195	(68.4)	0.199
	Out of Jazan region	23	(40.4)	34	(59.6)	
Previous knowledge about danger signs in neonatal illness	Yes	58	(29.3)	140	(70.7)	0.084
	No	55	(38.2)	89	(61.8)	
Received any information about the neonatal care	Yes	65	(26.5)	180	(73.5)	<0.001 *
	No	48	(49.5)	49	(50.5)	
Practice in any type of neonatal care during college training	No	27	(35.5)	49	(64.5)	0.601
	Yes	86	(32.3)	180	(67.7)	
Recall personally an ill neonate with any of the neonatal danger signs during study or practice	No	63	(34.8)	118	(65.2)	0.462
	Yes	50	(31.1)	111	(68.9)	
Previous knowledge about management of the very ill neonate	Yes	55	(30.9)	123	(69.1)	0.380
	No	58	(35.4)	106	(64.6)	
Overall level of knowledge		113	(33.0)	229	(67.0)	
95% CI For Good knowledge		(61.8–71.7)				

*p* value is based on Pearson chi-squared test and # *p* value is based on Fisher exact test; CI = confidence interval. * *p* value < 0.05 is considered statistically significant.

**Table 4:** Comparisons of mean knowledge scores according to some selected characteristics.

Variables		Knowledge Score (%)			Effect Size (η^2^)/Cohen’s d	F/t Value	*p* Value
		Number	Mean	SD			
Gender	Male	58	58.3	13.7	0.080	0.557	0.578
	Female	284	57.2	13.8			
Year of study in the faculty of nursing	2nd year	12	57.0	14.1	0.020	0.210	0.889
	3rd year	104	57.5	14.4			
	4th year	196	57.7	13.4			
	Internship year	30	55.5	13.9			
Marital status	Non married	276	58.4	13.8	0.029	2.55	0.039 *
	Married with children	23	52.9	12.5			
	Married without children	22	56.9	13.0			
	Divorced	6	51.0	12.9			
	Widow	15	49.6	13.9			
Area of residence	Jazan(urban)	182	57.2	13.2	0.016	2.7	0.069
	Jazan (rural)	103	59.4	13.3			
	Out of Jazan region	57	54.2	15.6			
Previous knowledge about danger signs in neonatal illness	Yes	198	58.6	13.6	0.207	1.888	0.060
	No	144	55.8	13.8			
Received any information about the neonatal care	Yes	245	59.3	13.6	0.50	4.148	<0.001 *
	No	97	52.6	13.1			
Practice in any neonatal care during college training	Yes	266	57.5	13.7	0.024	0.187	0.852
	No	76	57.1	14.1			
Recall an ill neonate personally with any of the neonatal danger signs during study or practice	Yes	161	57.3	12.3	0.015	0.020	0.887
	No	181	57.5	15.0			
Previous knowledge about the management of the very ill neonate	Yes	178	58.0	13.5	0.085	0.788	0.431
	No	164	56.8	14.1			
Attitude score	Poor	163	56.7	13.5	0.100	0919	0.359
	Good	179	58.0	14.0			

* *p* value < 0.05 is considered statistically significant.

**Table 5:** Binary logistic regression for the factors associated with students’ knowledge about neonatal danger signs.

Variables	B	S.E.	Wald	*p* Value	AOR	95% C.I. for AOR	
						Lower	Upper
Gender (Male)	0.23	0.34	0.44	0.507	1.25	0.64	2.45
Marital status (Single)	0.12	0.35	0.12	0.734	1.13	0.57	2.25
Area of residence (Jazan town)	0.30	0.31	0.89	0.345	1.34	0.73	2.49
Previous knowledge about danger signs in neonatal illness (Yes)	−0.11	0.31	0.11	0.735	0.90	0.49	1.66
Received any information about the neonatal care (Yes)	1.08	0.31	11.82	0.001	2.95	1.59	5.47
Practice in any type of neonatal care during college training (Yes)	−0.06	0.31	0.04	0.845	0.94	0.51	1.73
Recall personally an ill neonate with any of the neonatal danger signs during study or practice (Yes)	0.08	0.27	0.08	0.773	1.08	0.64	1.83
Previous knowledge about management of the very ill neonate (Yes)	0.21	0.30	0.48	0.487	1.23	0.68	2.22
Attitude score (%)	0.002	0.005	0.06	0.812	1.00	0.99	1.01

Abbreviations: CI = confidence interval; AOR = adjusted odds ratio, and SE = Standard error. *p* value < 0.05 is considered statistically significant.

## Data Availability

No new data were created or analyzed in this study.
